# The impact of frequently neglected model violations on bacterial recombination rate estimation: a case study in *Mycobacterium canettii* and *Mycobacterium tuberculosis*

**DOI:** 10.1093/g3journal/jkac055

**Published:** 2022-03-07

**Authors:** Susanna Sabin, Ana Y Morales-Arce, Susanne P Pfeifer, Jeffrey D Jensen

**Affiliations:** Center for Evolution and Medicine, School of Life Sciences, Arizona State University, Tempe, AZ 85281, USA

**Keywords:** *Mycobacterium canettii*, *Mycobacterium tuberculosis*, population genomics, genetic hitchhiking, Hill–Robertson interference, progeny skew, recombination rate estimation, LDhat

## Abstract

*Mycobacterium canettii* is a causative agent of tuberculosis in humans, along with the members of the *Mycobacterium tuberculosis* complex. Frequently used as an outgroup to the *M. tuberculosis* complex in phylogenetic analyses, *M. canettii* is thought to offer the best proxy for the progenitor species that gave rise to the complex. Here, we leverage whole-genome sequencing data and biologically relevant population genomic models to compare the evolutionary dynamics driving variation in the recombining *M. canettii* with that in the nonrecombining *M. tuberculosis* complex, and discuss differences in observed genomic diversity in the light of expected levels of Hill–Robertson interference. In doing so, we highlight the methodological challenges of estimating recombination rates through traditional population genetic approaches using sequences called from populations of microorganisms and evaluate the likely mis-inference that arises owing to a neglect of common model violations including purifying selection, background selection, progeny skew, and population size change. In addition, we compare performance when full within-host polymorphism data are utilized, versus the more common approach of basing analyses on within-host consensus sequences.

## Introduction

The causative agents of tuberculosis are immensely successful bacterial pathogens, maintaining a reservoir in ∼1.7 billion humans through latent infection (Houben and Dodd 2016). Such infections also resulted in the deaths of approximately 1.3 million HIV-negative and over 200,000 HIV-positive individuals in 2020, thus representing a critical public health concern (WHO 2021). Most of these organisms are members of the *Mycobacterium tuberculosis* complex (MTBC), which is composed of 9 human-associated lineages (lineages 1–4, 7, and 8: *M.* *tuberculosis* sensu stricto; lineages 5, 6, and 9: *Mycobacterium* *africanum*) and 1 group of animal-associated strains, including amongst others *Mycobacterium* *bovis* and *Mycobacterium* *microti* (Gagneux 2018; Ngabonziza *et al.* 2020; Coscolla *et al.* 2021). Despite the MTBC being a thoroughly studied family of pathogens relevant to human health, relatively little is understood about the evolutionary history and dynamics of these organisms.

A closely related organism, *Mycobacterium canettii*, generally considered an analog for the common ancestor of the MTBC (Gutierrez *et al.* 2005), is frequently used as an outgroup in phylogenetic analyses. Although it also causes tuberculosis in humans, *M. canettii* differs from the complex in several ways. The most notable differences for our purposes here include (1) the dramatically decreased genetic diversity in the MTBC relative to *M. canettii*, and (2) the occurrence of recombination in *M. canettii*, which is not thought to widely occur in the MTBC (Pepperell *et al.* 2013; Boritsch *et al.* 2016; Godfroid *et al.* 2018). Other peculiarities of *M. canettii* include its larger and more variable genome length (Gagneux 2018), its geographical isolation to the Horn of Africa (Gagneux 2018), the lack of apparent transmission between humans (Fabre *et al.* 2010; Koeck *et al.* 2011; Supply *et al.* 2013; Blouin *et al.* 2014), and the likelihood that it is maintained in an as-of-yet unidentified environmental reservoir (Aboubaker Osman *et al.* 2016). *Mycobacterium canettii*’s phylogenetic relationship to the MTBC, and its many intriguing differences from the complex, make it of clear interest for comparative studies.

In pairwise comparisons, 2 strains from the MTBC may differ by as many as ∼2,500 single nucleotide polymorphisms (SNPs), while 2 *M. canettii* strains may differ by up to 65,000 SNPs (Gagneux 2018). There is genetic (Supply *et al.* 2013; Mortimer and Pepperell 2014) and experimental (Boritsch *et al.* 2016) evidence that *M. canettii* undergoes a form of horizontal gene transfer—distributive conjugal transfer (DCT)—in which tracts of unlinked donor DNA of variable size and location are transplanted into a recipient bacterial genome (Gray and Derbyshire 2018). Though some work has identified evidence of highly limited recombination in parts of the genome difficult to resolve with short-read sequencing technologies (Liu *et al.* 2006; Namouchi *et al.* 2012), the majority of the literature agrees that the MTBC is functionally clonal (Pepperell *et al.* 2013; Chiner-Oms *et al.* 2019). Furthermore, organisms in the MTBC are missing the “mating identity” (*mid*) genes that appear essential for DCT in *M. canettii* (Gray and Derbyshire 2018). In addition, there is no evidence for recombination between contemporary MTBC and *M. canettii* strains, though evidence has been presented that the common ancestor of the MTBC may have shared an ecological niche and recombined with ancestral *M. canettii* strains (Chiner-Oms *et al.* 2019), and recent in vitro experiments found that it is possible for MTBC bacilli to act as DNA donors to *M. canettii* recipients (Madacki *et al.* 2021).

Previous studies have investigated aspects of the evolutionary genomics of MTBC compared to other *Mycobacteria*. This has included the calculation of the ratio of nucleotide substitution rates at nonsynonymous and synonymous sites (*d_n_*/*d_s_*) as well as a quantification of the site frequency spectra (SFS) from MTBC patient data, revealing an excess of low-frequency variants relative to standard neutral Wright–Fisher expectations, which has been attributed to purifying selection effects (Pepperell *et al.* 2010, 2013; Brown *et al.* 2016; Lieberman *et al.* 2016). More recently, [Bibr jkac055-B67][Bibr jkac055-B67][Bibr jkac055-B67] fit a more comprehensive evolutionary null model to within-host MTBC data (generated by Trauner *et al.* 2017), demonstrating that, in addition to purifying and background selection (Charlesworth *et al.* 1993, 1995), progeny-skew (i.e. a large variance and skew in progeny number) and infection bottlenecks also act to shape genomic variation in important ways (see Irwin *et al.* 2016; Matuszewski *et al.* 2018; Sackman *et al.* 2019; Jensen 2021; Morales-Arce *et al.* 2021). Thus, a variety of non-neutral and nonequilibrium processes appear to contribute to the observed level and distribution of genomic variation.

With these null processes now better quantified, it is of interest to evaluate whether the presence/absence of recombination in *M. canettii*/MTBC may itself (at least in part) explain the striking differences in observed levels of genomic heterogeneity between the two. The evolutionary advantage of recombination in breaking linkage between sites, thereby allowing natural selection to more efficiently maintain beneficial variants and purge deleterious ones by uncoupling them from one another, has been long-appreciated (Fisher 1930; Muller 1932; Hill and Robertson 1966). This so-called Hill–Robertson interference amongst sites has important implications, not only for the probabilities of fixation and loss (Hill and Robertson 1966; Maynard Smith and Haigh 1974; Haigh 1978; Charlesworth *et al.* 1993; Charlesworth 1994; Gordo and Charlesworth 2000; see review of Charlesworth and Jensen 2021) but when combined with genetic drift and mutational pressure may also lead to the “clicking” of Muller's Ratchet (i.e. the periodic and irreversible loss of the fittest class of individuals; Muller 1964; Felsenstein 1974). This mechanism—the speed of which is modulated by mutation rate, effective population size, and the strength of selection acting upon deleterious mutations—may lead to extinction in nonrecombining populations (Lynch *et al.* 1993; Bank *et al.* 2016; Matuszewski *et al.* 2017; Jensen 2021).

**Fig. 1.**
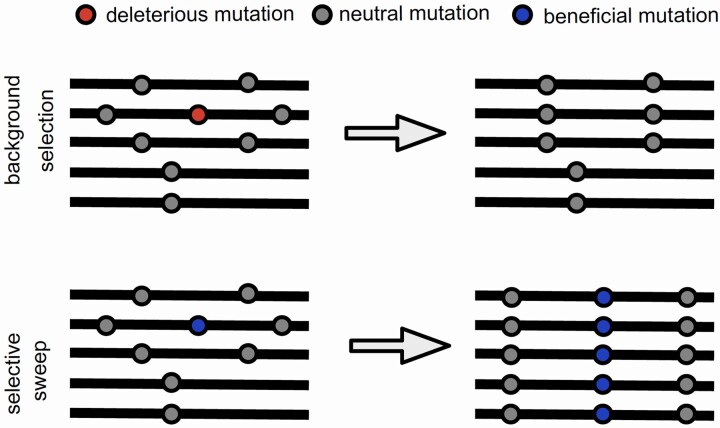
Graphical example of Hill–Robertson effects. Hitchhiking effects associated with linkage to a deleterious mutation, known as background selection, may act to reduce variation, as purifying selection acting on the deleterious mutation (shown in red) may result in the elimination of linked variants. Similarly, hitchhiking effects associated with linkage to a beneficial mutation, known as a selective sweep, may also act to reduce variation, as positive selection acting on the beneficial mutation (shown in blue) may result in the fixation of linked variants.

Background selection is one important realization of this interference, in describing the effects of linkage to deleterious variants (Charlesworth *et al.* 1993), as is another type of genetic hitchhiking, selective sweeps, in describing the effects of linkage to beneficial variants (Maynard Smith and Haigh 1974) (Fig. 1). The magnitude of the effects of these processes, both in their resulting reductions in genomic variation and local effective population size, will naturally be greater as the rate of recombination is reduced (i.e. reduced recombination leads to greater genomic linkage). In the absence of recombination, as in MTBC, the elimination of a deleterious variant, as well as the fixation of a beneficial one, is thus expected to have long-range genomic effects. Importantly, while background selection effects will always be more pervasive than selective sweeps owing to the much larger mutational input of deleterious relative to beneficial variants (see reviews of Eyre-Walker and Keightley 2007; Bank *et al.* 2014 ), this disparity is only expected to be amplified in the absence of recombination owing to the reduced probability of fixation of beneficial variants. If a beneficial mutation does sweep to fixation in this context, it will often carry with it linked deleterious variants, thus increasing the fixation load. In sufficiently small populations that do not recombine, the accumulation of deleterious variants alone can lead to population extinction (Lynch and Gabriel 1990; Lynch *et al.* 1993; Jensen and Lynch 2020). In addition, it has been demonstrated that with high enough mutation rates, beneficial fixation probabilities can approach zero owing to this deleterious linkage (Pénisson *et al.* 2017). In these ways, a lack of recombination may limit adaptive potential, and can do so in a compounding way.

This reduced adaptive potential has been well-studied within the context of viruses, *Drosophila*, and plants. Experimental in vitro studies on the effect of the broad-spectrum, mutagenic drug favipiravir on influenza A virus have empirically demonstrated the potential disastrous effects of accelerated mutation in nonrecombining asexual populations, including a reduction in effective population size, the accumulation of mutational load, population decline, and ultimately extinction (Bank *et al.* 2016; Ormond *et al.* 2017). *Drosophila* populations have also been found to experience a reduced efficacy of selection and capacity for adaptation in genomic regions with low recombination rates (Betancourt and Presgraves 2002; Haddrill *et al.* 2007), and the genetic degeneration and extinction seen in Y chromosomes have been largely attributed to Hill–Robertson effects following the shut-down of recombination (Bachtrog 2013). Comparisons of selfing vs out-crossing species have observed similar effects (Bustamante *et al.* 2002; Arunkumar *et al.* 2015). Furthermore, empirical evidence has provided support for the notion that Hill–Robertson effects cannot be entirely avoided, even in genomic regions with high recombination rates (Charlesworth 2009).

For these many reasons, the ability to accurately characterize recombination rates in natural populations—particularly for human pathogens—is crucial. In the case of the MTBC, with complete functional clonality, these described effects would be expected to be particularly severe, but may be largely mitigated in *M. canettii*. Given these expectations from population genetic theory, we here examine the role of recombination in shaping genomic diversity in *M. canettii* and the MTBC. In order to do so, we provide some of the first estimates to date of within-host diversity from *M. canettii* sequencing data, and generate the first statistical estimates of the rate of recombination. Through comparing within-host (i.e. full polymorphism data) and between host (i.e. consensus sequence) diversity in empirical and simulated *M. canettii* data, we also demonstrate inherent limitations to the widely used consensus sequence approach for microbial populations. In addition, we illustrate the confounding effects of non-Wright–Fisher population dynamics on the estimation of population-level statistics. Specifically, we quantify the roles of population size change, progeny skew, and purifying and background selection in biasing recombination rate estimation. While contextualized within the Mycobacterium as an example, these concerns are broadly applicable across many human pathogens.

## Materials and methods

### Data collection

Whole-genome sequencing data from patient isolates of *M.* *canettii* was downloaded from the NCBI sequencing read archive (SRA). The samples were limited to those attached to publications, such that we were able to readily access contextual information about the collection and sequencing methods. This search, conducted in January 2020, yielded a total of 18 *M. canettii* isolates from 18 patients from Djibouti, France, and the United States (Fabre *et al.* 2010; Blouin *et al.* 2012, 2014; Shea *et al.* 2017). To compare within-host variation between *M. canettii* and the *M.* *tuberculosis* complex (MTBC), a dataset of comparable size was assembled from MTBC sequencing data (Comas *et al.* 2013, 2015; Orgeur *et al.* 2021) spanning the known diversity of the complex (Supplementary Table 1), barring the recently identified lineages 8 and 9 (Ngabonziza *et al.* 2020; Coscolla *et al.* 2021).

### Alignment and within-host variant calling

*Mycobacterium canettii* and MTBC sequencing data was quality-checked using FastQC v. 0.11.7 (Andrews 2010) and processed according to a previously reported pipeline for within-host variant calling, based on the methods of Trauner *et al.* (2017). In short, raw sequencing data was preprocessed using scythe v. 0.994 (Buffalo 2020) and sickle v. 1.33 (Joshi and Fass 2011) for Bayesian and quality-based adapter trimming. Alignments were performed using BWA *aln* v. 0.7.17 (Li and Durbin 2009) using CIPT 140010059 (GCA_000253375.1) and H37Rv (GCF_000195955.2) as reference for the *M. canettii* and MTBC samples, respectively. Next, alignments were sorted, merged, and indexed with SAM tools v. 1.9 (Li *et al.* 2009; Danecek *et al.* 2021) and duplicate reads removed using Picard v. 2.9.2 (Broad Institute 2017). In addition, indel realignment and base quality recalibration were performed using the Genome Analysis Toolkit (GATK) v. 3.7.0 (Van der Auwera *et al.* 2013; Van der Auwera and O’Connor 2020). For within-host variant calling, variants were called with both LoFreq* v. 2.1.3.1 (using the “holm” strand bias filter and requiring a coverage between 50 and 3,000 reads at variant sites; Wilm *et al.* 2012) and VarScan2 v. 2.3.9 (using the *mpileup2snp* tool and requiring a minimum coverage of 50, a minimum of 4 supporting reads in each direction, a minimum variant frequency of 0.005, and a “—strand-filter” of 1; Koboldt *et al.* 2012), retaining only sites that were congruent between the 2 tools. The base calls were summarized using SAM tools *mpileup*, with a minimum mapping quality of 30 and a minimum sequencing quality of 20. As the quality of the read data often deteriorates with increasing read length, SNPs near read ends were excluded using a Kolmogorov–Smirnov test, implemented as in Trauner *et al*. (2017). Moreover, repetitive regions can pose challenges for read alignment and can lead to spurious variant calls (Pfeifer 2017), thus these regions were removed from consideration. To this end, a list of problematic regions was acquired for MTBC (e.g. Bos *et al.* 2014; Lieberman *et al.* 2016). An equivalent list for *M. canettii* was assembled for the purpose of this study, containing regions analogous to the *M. canettii* genome (Supplementary Table 2). Specifically, a .bed file was generated based on annotations from the CIPT 140010059 (GCA_000253375.1) annotation .gff file containing regions of the PE-PGRS family protein, PE family protein, and PPE family proteins, as well as integrases, transposases, and prophages (Supplementary Table 3), which have been systematically excluded in MTBC studies (e.g. Comas *et al.* 2010).

### Alignment and between-host (consensus sequence) variant calling

To replicate variant calling from consensus sequences—as performed in the original studies which published the empirical patient data (Fabre *et al.* 2010; Blouin *et al.* 2014; Shea *et al.* 2017)—*M. canettii* data from patient isolates were aligned to the RefSeq representative sequence *M.* *canettii* CIPT 140010059 (GCA_000253375.1) using BWA *mem* v. 0.7.17 (Li and Durbin 2009) and sorted using SAM tools v. 1.9 (Li *et al.* 2009; Danecek *et al.* 2021). Qualimap v. 2.13 (Okonechnikov *et al.* 2016) was used to calculate mapping statistics for each sample and MultiQC v. 1.7 (Ewels *et al.* 2016) was used to visualize the results (Supplementary Table 4). To create congruence between the empirical and simulated data (for additional details, see “simulations”), it was necessary to down-sample the empirical data to a standardized depth of coverage across samples (i.e. a standardized number of individual genomes). To avoid a loss of data during the variant calling while retaining as many samples as possible, 25-fold was chosen as the standardized coverage, excluding ERR266117 (18-fold mean coverage) from further analysis. Specifically, the mean coverage of each sample’s alignment was down-sampled to 25-fold with SAM tools *view* v. 1.9 (Li *et al.* 2009; Danecek *et al.* 2021). Next, FreeBayes v. 1.1.0 (Garrison and Marth 2012) was used for variant calling, using a ploidy (-p) of 2 to allow multiple variants per site. A minimum of 2 supporting observations was required for each variant call to protect against the incorporation of sequencing errors. A minimum mapping quality of 60 was used, and the “—pooled-continuous” setting of FreeBayes was applied to treat samples as representative of bacterial populations rather than monoisolates. Following variant calling, VCFtools “—exclude-bed” v. 0.1.12 (Danecek *et al.* 2011) was used to remove indels as well as variants in repetitive elements from the resulting variant calling files (.vcf) using our list of problematic regions (for details, see “Alignment and within-host variant calling”). BWA and Freebayes parameters were specified to reflect those implemented in the Snippy pipeline (https://github.com/tseemann/snippy), which was found to be a well-performing SNP calling pipeline for bacterial genomes (Bush *et al.* 2020). Consensus calling was performed using the BCFtools v. 1.9 (Danecek *et al.* 2021) “consensus” command, calling the first allele in the FORMAT/GT field (bcftools consensus -H 1), and grafting variant sites onto the *M. canettii* representative sequence (see above).

### Recombination rate estimation

A global per-site recombination rate for *M.* *canettii* was estimated using the 17 patient-derived samples described above. Consensus sequences for the *M. canettii* patient data were concatenated into a single multi-fasta file to which a header was added (“17 4482059 1”) designating 17 sequences of length 4,482,059 bp with a ploidy of 1. This multi-fasta was converted using the LDhat v. 2.2 (McVean *et al.* 2002) “convert” tool to LDhat-compatible input (.*sites* and .*locs*) files which were used in LDhat “pairwise” to calculate the most likely value for the population-scaled recombination rate (*ρ  *= 2 *N_e_r*, where *N_e_* is the effective population size and *r* the recombination rate per site per generation). To speed up computations, the per-site Watterson’s *θ* calculated by LDhat was used to generate a likelihood lookup table, with a maximum *ρ* of 100 and a grid size of 201.

### Simulations

Violations of the population genetic model assumptions underlying LDhat can impact recombination rate estimates (Dapper and Payseur 2018). To test whether *M. canettii*'s specific population dynamics impact our estimates of recombination, 8 models with varying selection intensities and population parameters were simulated using SLiM v. 3 (Haller and Messer 2019). Models were based on the *M.* *tuberculosis* null model developed by [Bibr jkac055-B67][Bibr jkac055-B67][Bibr jkac055-B67], assuming that *M. tuberculosis* and *M. canettii* experience a similar course of infection. The models varied by 3 parameters: the distribution of fitness effects (DFEs), occurrence of a population bottleneck, and presence of progeny skew. The Wright–Fisher (Base) compatible model had only 1 class of neutral mutation, in which the selection coefficient (*s*) was 0, no bottleneck occurred, and progeny skew was absent. The remaining 7 models had at least 1 of the 3 parameters implemented (Table 1). Models with a non-neutral DFE had 1 class of nearly neutral deleterious mutation (*s* = –0.001) and 1 class of weakly deleterious mutation (*s* = –0.01), as implemented by Morales-Arce *et al.*(2020). In models with progeny skew, the degree of skew, *ψ*, was modeled at 0.067, based on a strategy presented by Sackman *et al.* (2019), and subsequently implemented by Morales-Arce *et al*. (2020). This *ψ* value was previously found to have the strongest posterior density for *M. tuberculosis* (Morales‐Arce *et al.* 2020). Last, models with a bottleneck (Bn) experienced a reduction in census size to 50 at generation 100,001, followed by a period of exponential population recovery spanning 90 generations, representing an infection bottleneck and subsequent growth.

**Table 1.** Summary of simulation models.

	Evaluated parameters		
Model	Bottleneck (Bn)	Progeny Skew (ψ)	Distribution of fitness effects (DFE)
Base	X	X	X
Base + Bn	✓	X	X
Base + Bn + DFE	✓	X	✓
Base + Bn + ψ	✓	✓	X
Base + Bn + ψ + DFE	✓	✓	✓
Base + DFE	X	X	✓
Base + ψ	X	✓	X
Base + ψ + DFE	X	✓	✓

“Base” represents the standard Wright–Fisher (WF) model, “Bn” a bottleneck, “DFE” the presence of non-neutral mutations, and “Ψ” a non-Wright–Fisher progeny skew (for simulation details, see *Materials and Methods*).

Simulations were implemented as “nucleotide” models in SLiM using a per-site per-generation mutation rate (*μ*) of 6.0 ×10^−8^ [i.e. the mutation rate inferred by Morales-Arce *et al.* (2020) for the MTBC], corresponding to a rate of 2.0 ×10^−8^ per possible nucleotide transition per site in a Jukes-Cantor mutation model, and a per-site per-generation recombination rate of 7.2 ×10^−11^ (in accordance with our estimate; see “*Results and Discussion*”). Each model had a starting census size (*N*) of 10,000 individuals and, to reduce computational burden, a genome length of 413, 587 bp (i.e. 10% of the full length of the *M.* *canettii* representative genome, minus the repetitive regions we chose to exclude). One hundred replicates were run for each model, using a burn-in of 100,000 generations (i.e. 10N) and a total of 101,000 generations. SLiM v. 3 assumes diploidy in its simulations and models mutations as occurring on a “genome1” and “genome2” for each individual. To enforce haploidy, all mutations occurring on the “genome2” for each individual were excluded, and mutations with frequency 0.5 or greater were treated as fixed mutations rather than variant sites. From each replicate simulation, a subset of 500 individuals was selected from the last simulated generation, and the “genome1” sequence from each individual was included in a multi-fasta file.

To test the performance of LDhat v. 2.2 under different model violations, “pairwise” was run on the simulated data (following the method outlined in “Recombination rate estimation”) to estimate the population-scaled recombination rate. Specifically, to replicate both (i) within-host diversity and (ii) between-host (consensus) calling from the bacterial population, datasets of (i) 100 sequences and (ii) 17 sets of 25 sequences from the 500 sequences simulated per model replicate (corresponding to 17 empirical bacterial populations with 25-fold genomic coverage) were randomly selected, respectively (Supplementary Fig. 1), and rudimentary consensus sequences were called based on the most common allele at each site from each set of 25 sequences for the latter. The custom python scripts used in these analyses are available on GitHub (see “*Data Archiving*”).

## Results and discussion

### Within-host variation in *M. canettii* and the *M. tuberculosis* complex

**Fig. 2.**
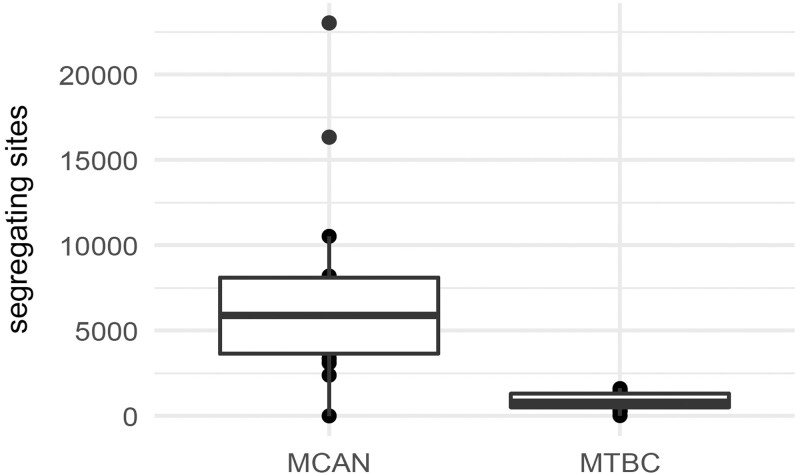
Comparison of within-host variation between *M. canettii* and MTBC. *Mycobacterium canettii* has both a greatly elevated mean and variance in genome-wide segregating sites relative to the MTBC.

We utilized whole-genome sequencing data of *M.* *canettii* from clinical and public health projects in Djibouti, France, and the United States, containing 18 documented *M. canettii* isolates with isolation dates spanning from 1983 up to 2016 (Fabre *et al.* 2010; Blouin *et al.* 2014; Shea *et al.* 2017). In addition, we assembled a comparable dataset for the *M.* *tuberculosis* complex from published MTBC sequencing data (Comas *et al.* 2013, 2015; Orgeur *et al.* 2021), with isolates representing the majority of the known lineages. Segregating sites within each isolate were identified through a pipeline modified after Trauner *et al*. (2017), which applies 2 low-frequency variant callers to the sequencing data, followed by stringent variant filtering (see “*Materials and Methods*” for details). Compared to the MTBC dataset, the *M. canettii* dataset contains many more segregating sites within isolates (MTBC median: 810, mean: 845; *M. canettii* median: 5,799, mean: 6,724) as well as a much wider variance across isolates (MTBC range: 19–1,607; *M. canettii* range: 0–23,030) (Fig. 2).

This result provides an additional dimension to the distinctions between *M. canettii* and the MTBC, in that the difference in diversity between the two extends beyond pairwise differences between consensus sequences from different isolates as previously reported, and into the within-host populations themselves. Thus, while there are contextual differences between the two types of bacteria, and while there may be differences in virulence and/or infection behavior, there is also a fundamental biological difference in the evolutionary dynamics governing these two organisms. Given our current understanding of their biology, it is likely that recombination is a dominant mechanism enhancing the within-host variation, and therefore overall population diversity, of *M. canettii* in comparison to the MTBC. The consequence of this for the MTBC—reduced efficacy of selection, reduced effective population size, and genomic degeneration—can be studied through the lens of Hill–Robertson interference.

The benefit to the MTBC as a group of obligate pathogens is the consistent retention of fundamental virulence traits (e.g. *phoR* gene as proposed by Chiner-Oms *et al.* 2019). This itself was likely at the cost of becoming an obligate pathogen therefore unable to survive independent of a host cell. Limited recombination potential, specifically the potential to accept donor DNA, likely enforced a program of genomic reduction (as seen in, for example, nonrecombining sex chromosome evolution; Bachtrog 2013). This is illustrated by the reduced and less variable pan-genome size in the MTBC as compared to *M. canettii*, and the obligate pathogenicity of the MTBC is in contrast to the seeming environmentally opportunistic pathogenicity of *M. canettii* (Aboubaker Osman *et al.* 2016).

### Recombination rate estimation in *M. canettii*

**Fig. 3.**
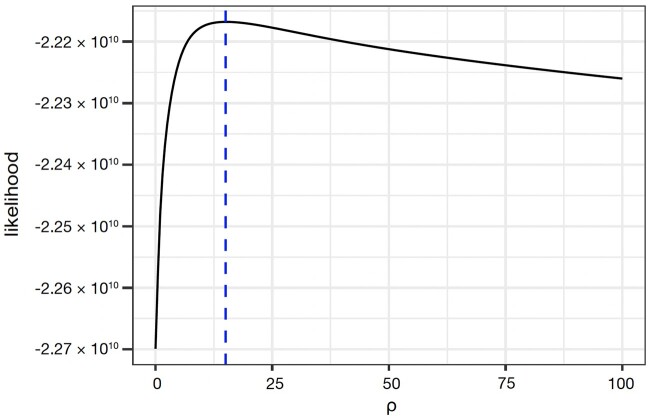
Distribution of likelihoods across different values of ρ for empirical *M. canettii*. Distribution of likelihood for different population-scaled recombination rates, ρ, ranging from 0 to 100 obtained using LDhat (see *Materials and Methods* for details). The blue line indicates the maximum likelihood at ρ = 15.

As the presence of recombination in *M. canettii* populations and lack thereof in MTBC populations may be a crucial factor in understanding the observed differences in within- and between-host diversity between the 2 bacterial groups, we used LDhat (McVean *et al.* 2002) to estimate the population-scaled recombination rate (*ρ *= 2 *N_e_r*, where *N_e_* is the effective population size and *r* the recombination rate per-site per-generation) in *M. canettii*. As LDhat requires an input of discrete genetic sequences, a consensus sequence was called for each *M. canettii* isolate. To facilitate standardized variant sampling across the empirical and simulated data, we subsampled patient sequences to a uniform mean coverage of 25-fold, representing approximately 25 individual genomes within an *M. canettii* population (Supplementary Fig. 1). This level of coverage was intended to strike a balance between including as much of the empirical dataset as possible, while maintaining sufficient coverage to confidently call variants. There were a total of 43,332 sites segregating amongst the consensus sequences, which were used to estimate a most likely value of *ρ* at 15 (Fig. 3). Assuming *M. canettii* and the MTBC have identical per-site per- generation mutation rates (*μ*), where,

$$\theta =\text{2}N_{e}\mu L,$$

we can calculate *r* based on the equivalency of ρ/θ and *r*/μ. Using *μ*  =  6.0 ×10^−8^ as estimated by Morales-Arce *et al*. (2020), this calculation yielded an estimate of *r *=* *7.2 ×10^−11^ for *M. canettii*.

### Fitness effects and population dynamics influence recombination rate inference

**Fig. 4.**
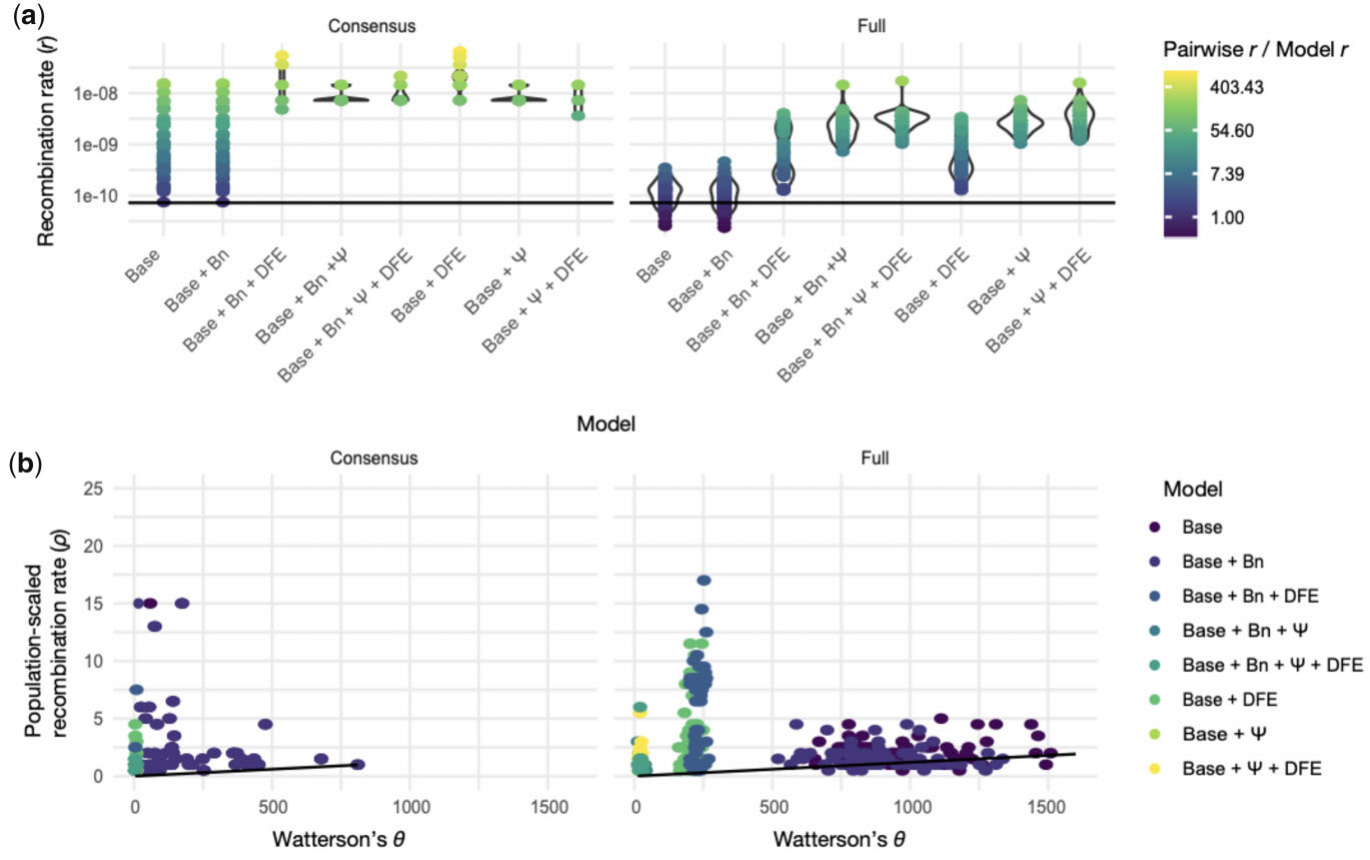
Performance of LDhat under a variety of model violations. a) the per-site recombination rate (*r*) inferred from ρ estimated by LDhat deviates strongly from the true value of *r* = 7.2 × 10^−11^ (see solid black horizontal line) in most models. Indeed, the correct estimate is obtained only when using full population data (rather than consensus data) under either the standard Wright–Fisher (Base) model or a neutral bottleneck model (Base + Bn). Each point corresponds to a simulated replicate, with replicates binned according to their model along the *x*-axis. The point color corresponds to the ratio between the LDhat-inferred *r* and the true *r* (log-scaled to improve visualization). b) Similar to panel a, the relationship between ρ and θ deviates from the slope of *r*/μ (solid black line) for many of the simulated model violations (colors represent different models). Both the standard Wright–Fisher (Base) and the neutral bottleneck (Base + Bn) models largely conform to expectation when the full population data are used. However, the use of consensus sequences as well as the presence of either natural selection and/or progeny skew, can cause extreme deviations from the true value.

Morales-Arce *et al.* (2020) recently presented an evolutionary null model for MTBC which included a population bottleneck associated with infection as well as reproductive progeny skew. As a causative agent of tuberculosis along with the MTBC, it is likely that *M. canettii* violates Wright–Fisher life history assumptions in a similar manner. To test the impact of potential model violations on our recombination rate estimates, we designed 8 different models in SLiM (Haller and Messer 2019) with varying levels of fitness effects, occurrence of population bottlenecks, and presence of progeny skew (Table 1). For all models, the true simulated recombination and mutation parameters were *r *=* *7.2 ×10^−11^ and μ = 6 0.0 ×10^−8^ per-site per-generation. The Wright–Fisher “Base” model represents a model with 1 class of neutral mutation and no bottleneck or progeny skew. Successive models consisted of the “Base” model with the addition of mildly deleterious mutations, a bottleneck, and progeny skew, individually or in combination. From the resulting simulated populations, we modeled the population variant sampling that took place during the generation of the *M. canettii* consensus sequences for the initial recombination rate estimate by taking 17 subsamples of 25 individual genomes from each simulated population (Supplementary Fig. 1). From these subpopulations, we performed rudimentary consensus calling, and performed LDhat analyses in parallel with the full populations. The *ρ* estimates for the “Base” model and “Base + bottleneck” (Bn) model (Table 1), led to the closest estimates of *r*, but the inference for most models deviated strongly from the true *r* (7.2 ×10^-11^ per-site per-generation) (Fig. 4a). These deviations were greatly exacerbated when the simulated populations were represented by consensus sequences (Fig. 4a). Upon considering the expected relationship between *ρ* and *θ*, it appears that the reduction of diversity in non-Wright–Fisher models is compensated for by a higher *ρ* estimate, rather than a reduced *N_e_*, which in turn serves to inflate inferred values for *r* (Fig. 4b).

LDhat was chosen to illustrate the effects of these various model violations, as it is one of the most widely used recombination rate estimators in the field. While other approaches have recently been proposed within the context of studying bacterial populations, they importantly rely on the same underlying assumptions, and thus the results and biases discussed here will likely be commonly observed across approaches. For example, Garud *et al.* (2019) based their recombination rate estimation on the observed decay of LD in their study of bacteria sampled from the gut microbiome. However, progeny skew is similarly neglected in their model (despite likely being of great relevance in their studied organisms). Because skewed progeny distributions are known to greatly impact observed LD (Eldon and Wakeley 2008)—in some cases generating very strong LD despite frequent recombination, while in other cases weak LD despite infrequent recombination—this parameter will be a critical consideration and component for any LD-based recombination rate estimator. Relatedly, the approach of Sakoparnig *et al.* 2021 similarly neglects such skews via their use of the Kingman coalescent—which assumes a small mean and variance of progeny number. Furthermore, their approach assumes that all mutations are selectively neutral (and unlinked to selected sites)—an assumption that will be strongly violated in coding-dense genomes such as those of many bacteria. For these reasons, as with LDhat, the simulation analyses presented here studying the effects of these model violations will be of importance across methodologies.

### Consensus calling from microbial populations obfuscates true diversity and biases inference of recombination rate

**Fig. 5.**
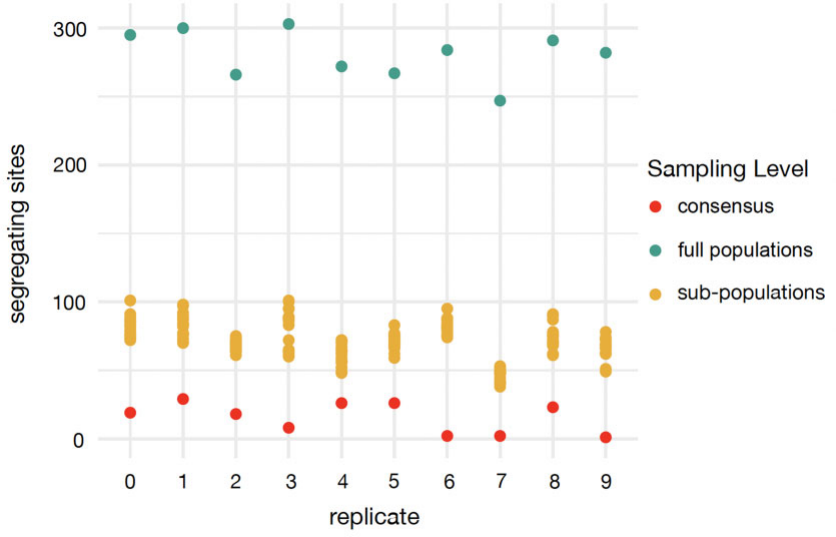
Segregating sites in consensus sequences and source populations, from simulated replicates. The number of segregating sites observed within each consensus, full population, and subpopulation dataset—for 10 simulation replicates of the ‘Base’ model with the addition of a bottleneck, progeny skew, and presence of non-neutral mutations (Base + Bn + Ψ + DFE). The full populations are represented by 500 genomes, the subpopulations are represented by 17 subpopulations consisting of 25 individual genomes each, and the consensus sequences are called for each subpopulation from the 25 constituent individual genomes. The points are binned by simulation replicates.

For many microbial isolates in clinical and environmental contexts, it is common practice to call a consensus sequence from an isolate, and many analytical tools assume a monoclonal isolate. To better understand the degree to which consensus sequences may represent their source populations, we explored the segregating sites found within the datasets that contribute to each level of consensus sequence building. We used a model with a population bottleneck, progeny skew, and presence of non-neutral mutations (Base + Bn  +  *ψ* + DFE) as a representative simulation model, as it is the most realistic model for *M. canettii*, and extracted 10 replicates. The diversity represented by the full population sampled from each replicate (*n *=* *500) was dramatically reduced when subsampled to a set of 17 subpopulations of 25 individuals each, corresponding to a collection of 17 isolates with genomic data with 25X coverage (Fig. 5; Supplementary Fig. 1). Moreover, the calling of consensus sequences from isolates greatly reduces observed diversity (Fig. 5)—an ascertainment that can greatly bias subsequent population genomics analyses as shown in Fig. 4 with regards to recombination rate estimation, and as previously demonstrated with regards to the inference of selection as well (Renzette *et al.* 2017).

## Conclusions

As the closest relative to the MTBC and a tuberculosis-causing bacterium, *M. canettii* is an intriguing outgroup for better understanding the evolutionary history of the MTBC and the emergence of tuberculosis as a global human disease. However, many of the ecological and biological characteristics underlying *M. canettii* remain poorly explored. Here, we examined the difference in diversity between *M. canettii* and MTBC species through the lens of recombination, linkage, and Hill–Robertson effects, and we explored methodological barriers that currently prevent a deeper understanding of these relationships.

Though we inferred a relatively low per-site per-generation recombination rate of 7.2 × 10^−11^ for *M. canettii*, our simulation-based power analyses considering possible violations to a Wright–Fisher model, as well as the nature of the empirical consensus-sequence-based data, suggest that this is likely an overestimate. The inference biases described here highlight the general importance of directly modeling consensus sequence construction prior to inference and power analyses. Specifically, by modeling the reduction in variation and change in frequency spectrum expectations as presented here (Figs. 4 and 5), one may quantify the extent to which subsequent analyses may be impacted when based upon consensus sequences, and hence how to better interpret results. Moreover, our analyses demonstrate that the consensus calling approach frequently applied in studies of complex microbial populations is far from ideal for estimating population genetic statistics and thus for inferring evolutionary parameters, and can greatly reduce the true diversity of a population by more than an order of magnitude. Although reconstructing individual genomes or haplotypes is challenging with most short-read sequencing technologies, highly accurate single-molecule long-read sequencing techniques (such as PacBio's SMRT sequencing) offer a powerful alternative to characterize the diversity of microbial populations.

It is important to note that the empirical data analyzed was sparse in the case of *M. canettii*, and that the available data was not generated with population genomic analysis in mind for either the *M. canettii* or MTBC datasets. As such, the clinical isolates may not be representative of the full distribution of diversity to be found in *M. canettii*, especially given that we have no environmental or “source” isolates. In addition, the represented isolates were cultured prior to whole-genome sequencing, thus imposing an additional bottleneck and limiting the discoverable diversity within the populations studied. It follows that, while the recombination rate estimated here for *M. canettii* is likely overestimated given the data, it is also seemingly the case that the full diversity of *M. canettii* populations is currently underestimated in the literature. Also, the simulations performed here accept a proposed MTBC null model (Morales‐Arce *et al.* 2020) as an analog for *M. canettii*. If the MTBC model is indeed comparable to that of *M. canettii*, *M. canettii*’s ability to accept donated chromosomal DNA (Madacki *et al.* 2021) is the most likely cause of its increased inter- and intra-isolated diversity compared to the MTBC. However, if the course and mode of infection differs in *M. canettii*, there may be other factors to consider. With these limitations in mind, to gain a more realistic sense of full *M. canettii* diversity, it is essential to identify the source of new infections and conduct in vitro experiments and additional genomic investigations of *M. canettii* infections to further interrogate the life history, infection course, and recombination frequency. Furthermore, it will be important to follow recommended population genomic practices that have been suggested for MTBC populations, in order to study within-host *M. canettii* diversity. These would include time-sampling of *M. canettii* populations from patients with active infections, deep whole-genome sequencing with the goal of identifying low-frequency variants, and the construction of a realistic evolutionary null model specifically for *M. canettii* (Morales-Arce *et al.* 2021).

Finally, in addition to other recent work studying the significant biases in recombination rate estimation that may arise from neglected nonequilibrium demographic histories (Dapper and Payseur 2018), our results emphasize the important and frequently neglected contributions of progeny skew as well as purifying and background selection. Future method development incorporating these various evolutionary processes would thus be of great use to the field, particularly for the study of human pathogens which are often characterized by extreme infection dynamics, a large progeny variance, and genomes that are strongly functionally constrained. Given the large number of parameters concerned, approximate Bayesian approaches are appearing the most promising for such future method development (e.g. Johri *et al.* 2020, 2022).

## Data availability

Custom scripts are available at https://github.com/sjsabin/mcan_popgen. The raw sequencing data are available via NCBI's Sequencing Read Archive (see Supplementary Table 1).

Supplemental material is available at *G3* online.

## Supplementary Material

jkac055_Supplementary_Figure_1

jkac055_Supplementary_Table_1

jkac055_Supplementary_Table_2

jkac055_Supplementary_Table_3

jkac055_Supplementary_Table_4
