# Supplementary material for: The impact of frequently neglected model violations on bacterial recombination rate estimation: a case study in Mycobacterium canettii and Mycobacterium tuberculosis
Source: G3 (Bethesda). 2022 Mar 7;12(5):jkac055. doi: 10.1093/g3journal/jkac055 (PMC9073693; doi:10.1093/g3journal/jkac055)
Supplement: jkac055_Supplementary_Figure_1 [file jkac055_supplementary_figure_1.docx]

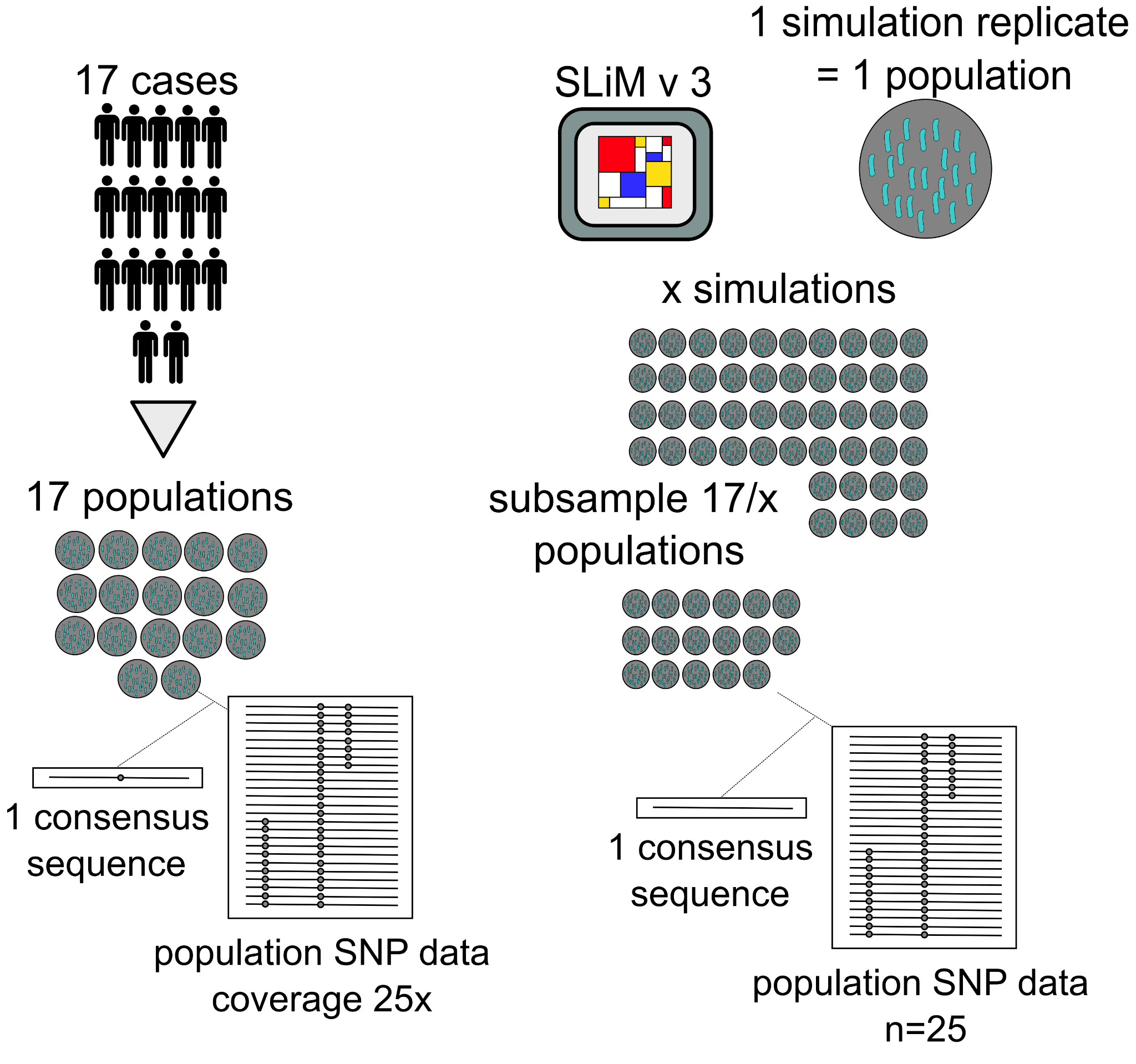


**Supplementary Figure 1.** Schematic representation of empirical and simulated data treatment to approximate equivalency between the datasets. As shown, for comparison with the 17 isolates sequenced at 25X coverage (left column), in the simulated data we sub-sampled 17 (within-host) populations from 25 individuals each (right column).
